# Achenbach Syndrome: A Case Series

**DOI:** 10.7759/cureus.22824

**Published:** 2022-03-03

**Authors:** Azin Azarfar, Shazia Beg

**Affiliations:** 1 Division of Rheumatology and Clinical Immunology, University of Florida, Gainesville, USA; 2 Rheumatology, University of Central Florida College of Medicine, Orlando, USA

**Keywords:** skin lesions, good prognosis, rare, benign, blue finger, achenbach syndrome

## Abstract

Achenbach syndrome, also known as "paroxysmal finger haematoma", is a rare, benign, self-limiting condition with unknown etiology that results in an acute onset swelling and pain, and subsequently blue discoloration of the fingers and sometimes the feet. The pathophysiology of this syndrome is not entirely clear, but intermittent spontaneous hematoma formation is reported as its characteristic symptom. Achenbach syndrome is more predominant in the female population. There are no known risk factors such as trauma, drug use, bleeding disorders, or rheumatologic diseases associated with the etiology of this syndrome. Although the symptoms are alarming to patients, the condition itself is not accompanied by any significant complications.

Herein we present our case series of four patients experiencing symptoms compatible with the diagnosis of Achenbach syndrome. The aim of this study is to increase awareness of this condition and its benign nature to avoid unnecessary referrals or invasive procedures and investigations as well as alleviate the anxiety of patients.

## Introduction

Achenbach syndrome, also known as “acute idiopathic blue finger”, or “paroxysmal finger hematoma”, is a rare (fewer than 100 cases have been reported), benign clinical disorder in which patients experience sudden onset painful swelling of their digits, with an ecchymosis-like discoloration [1]. It was first described by a German physician named Walter Achenbach in 1958 [2]. In his original work, he described six cases of female patients with "paroxysmal hand hematoma" or "finger apoplexy" who experienced recurrent attacks of acute onset pain on the volar aspect of one or more of their digits, followed by blue discoloration [2].

The etiology of the disease is not yet established. There is no reported association with trauma, occupation, exposure to warm or cold temperature, or body habitus. Increased vascular fragility, likely in the setting of minor trauma, causing capillary micro-haemorrhages has been proposed as the possible causation of the disease, although, many patients develop the condition with no identifiable trigger [1-4]. There have been no findings suggestive of thromboembolic or atheroembolic events, or vasculitic processes [5]. This syndrome is commonly seen in middle-aged female patients with a median age of 49.5 years [4]. Symptoms typically resolve spontaneously within days, without lasting sequelae [5]. 

## Case presentation

Case 1

**Figure 1:**
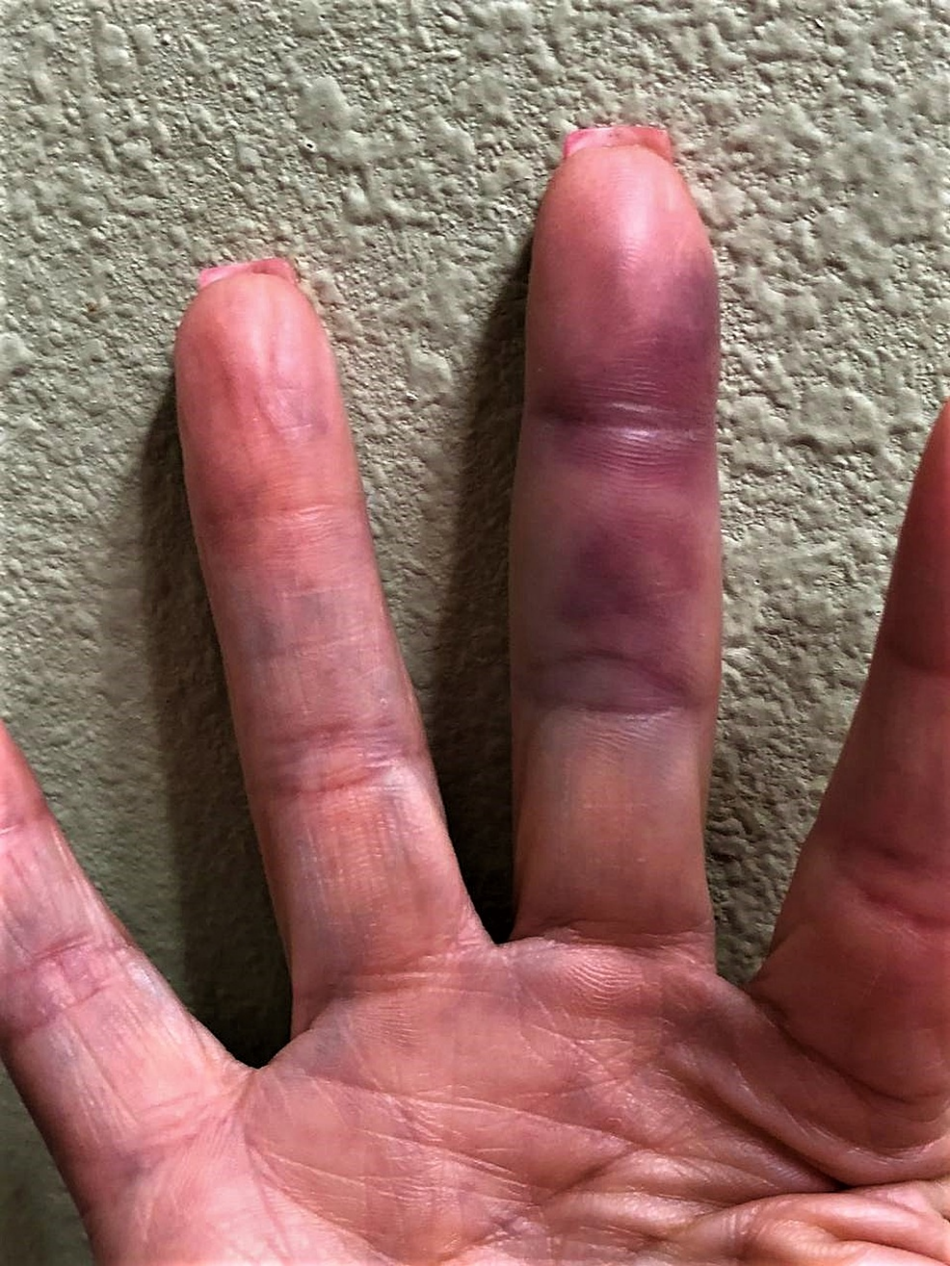
Blue discoloration of the middle finger

A 66-year-old Caucasian female with a past medical history of Sjogren’s syndrome (not on any medications), presented to the clinic for follow-up. Upon further questioning, she raised the concern about the new-onset purple, blue discoloration of some of her fingers. The discoloration was not correlated with exposure to cold temperature or any emotional distress. She endorsed that when the discoloration comes (two to three times per year), she feels some tingling sensation without experiencing any pain. These episodes subsided spontaneously within days, and without any treatment (Figure 1). Laboratory investigations showed a normal hematological, biochemical, and coagulation profile. On physical examination, all the extremities were warm, and the peripheral pulses were palpable.

Case 2

**Figure 2:**
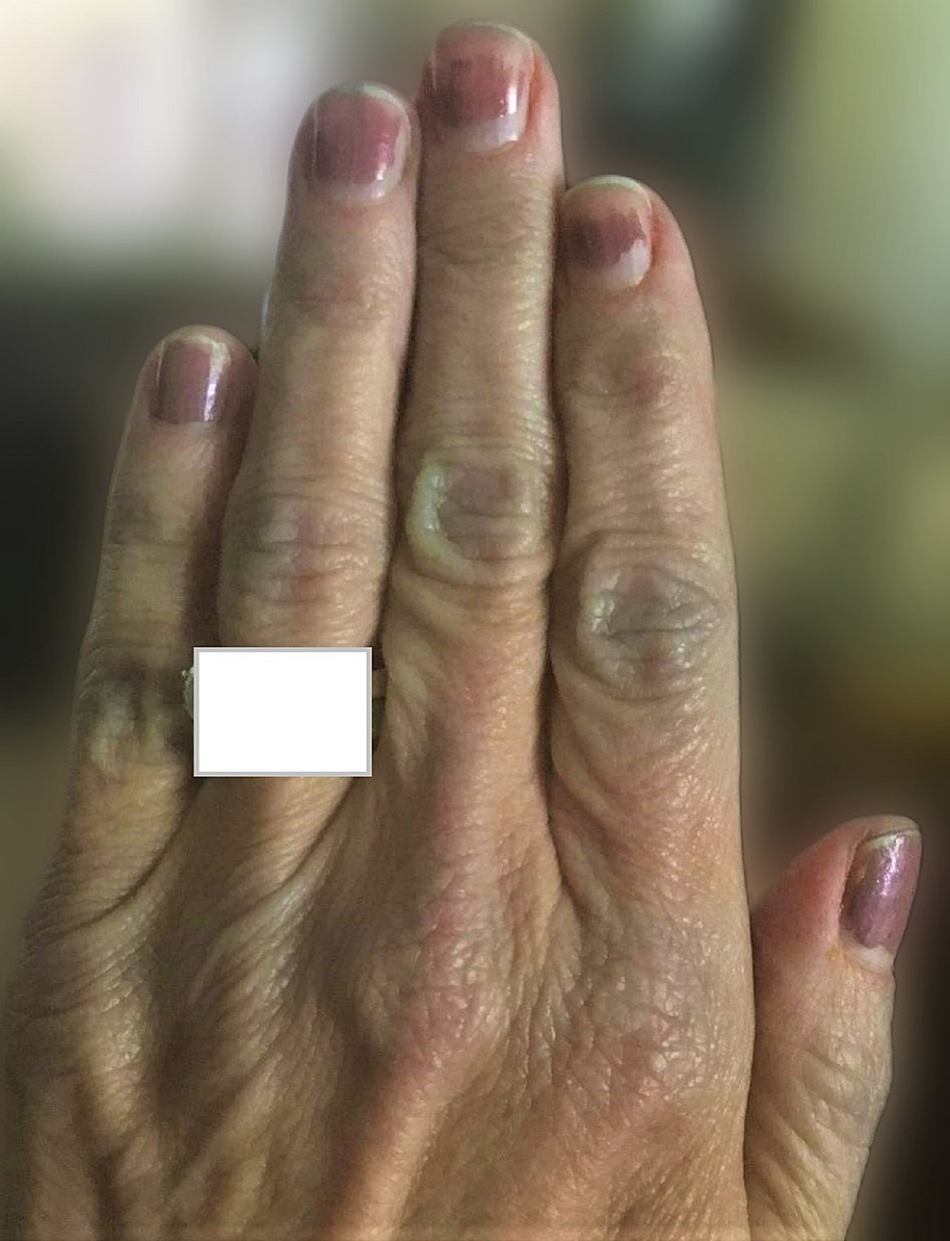
Blue discoloration of the knuckles

A 54-year-old Caucasian female with a history of seronegative rheumatoid arthritis and lupus, on abatacept, hydroxychloroquine and leflunomide, presented for follow-up. On review of systems, she described frequent episodes of mildly painful swelling with bluish discoloration over her knuckles. There was no association with a specific time of day, medications, trauma, or temperature changes. She did report that her teenage daughter has been experiencing similar symptoms which are mostly self-resolving. In her case, the discoloration subsided in a few days without any intervention (Figure 2). Her physical examination and laboratory findings were completely normal.

Case 3

**Figure 3:**
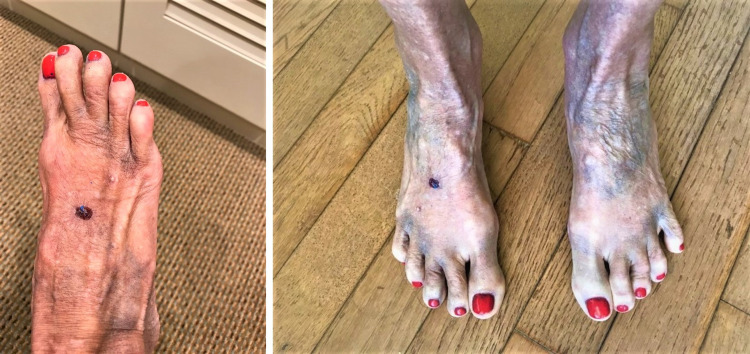
Blue discoloration scattered over the feet

A 64-year-old Caucasian female with a past medical history of systemic lupus erythematosus, well-controlled on hydroxychloroquine, visited the clinic presenting with recurrent episodes of sudden onset, painless, black-and-blue discoloration of her feet and toes. The patient did not report any correlation between her symptom with trauma, cold temperature, stress, or medications. The discoloration was preceded by a tingling sensation, followed by a change in the color of the skin one hour later. Most lesions disappear without any intervention within three days, however, some last longer, turning black and subsequently fading in a week (Figure 3). On exam, there was no evidence of joint synovitis or tenderness, and pedal pulses were normal.

Case 4

**Figure 4:**
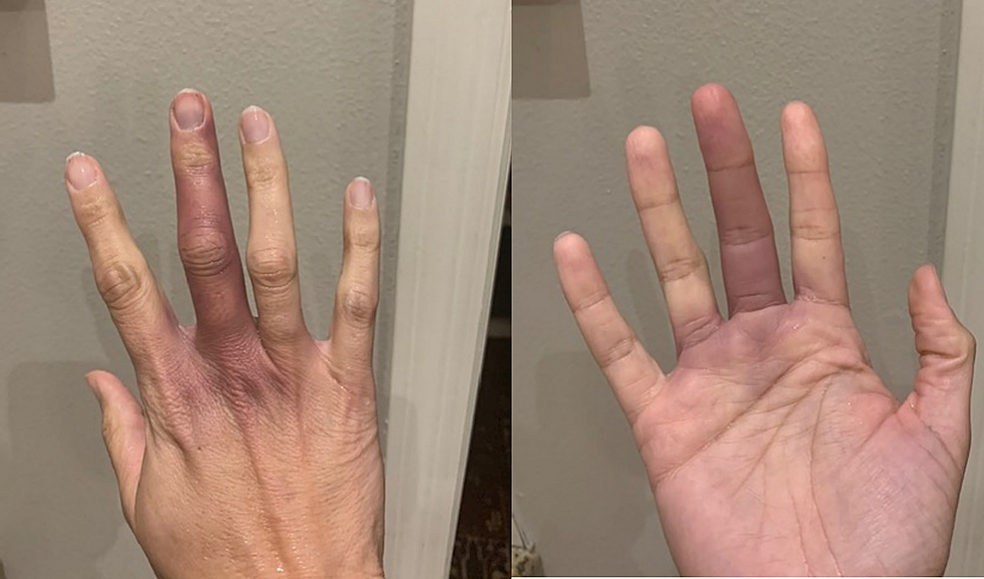
Blue discoloration of the middle finger

A 45-year-old Hispanic female with a history of seropositive rheumatoid arthritis, well-controlled on hydroxychloroquine, presented with acute onset finger swelling. She reported tingling in the third right digit, with gradual blue discoloration and swelling from blood pooling in it (Figure 4). There was no association with trauma or temperature change and the discoloration resolved in a few days. Laboratory studies showed no abnormalities in platelet count, coagulation panel, autoantibody, such as antinuclear antibody, and anti-phospholipid antibody. All the physical findings, including her joints, skin, and peripheral pulses were normal. The magnetic resonance imaging (MRI) of her hand did not show any abnormality.

## Discussion

Achenbach syndrome is a rare condition with unclear etiology. There are some reports of its correlation with acrocyanosis, gastrointestinal diseases, migraines, and biliary diseases [6]. All our patients in this case series had underlying rheumatologic conditions, however, there has been no report of any association of Achenbach syndrome with rheumatologic disorders.

Achenbach syndrome can affect both males and females, but the prevalence is higher in the female population [1,7]. It has been more observed in the volar surface of the fingers than the toes, involving most frequently the index followed by the middle finger [1,8].

Complete resolution of the symptoms usually occurs within a few days, although symptoms may last for a few months. Episodes can occur for a variable period with no sequelae.

Achenbach syndrome is mainly a diagnosis of exclusion and given its benign nature, no specific prevention and treatment have been proposed for it. Here are some of the important differential diagnosis that has been reported for Achenbach syndrome: Raynaud’s syndrome or phenomenon, spontaneous digital venous thrombosis, acute limb ischemia, collagen vascular disease, microemboli, Gardner-Diamond syndrome, and acrocyanosis [8,9].

Many patients with this condition are referred to rheumatologists, hematologists, or vascular surgeons and may undergo extensive and in some cases invasive investigations [9-11]. As this disease can be dreadful to patients and their family members, increasing awareness of its relatively benign nature can alleviate anxiety, and by providing reassurance we can avoid unnecessary interventions and testing [9-12].

A summary of previously published literature regarding Achenbach syndrome is shown in Table 1.

**Table 1:** Summary of the previous literature.

First Author, year	Patients Mean Age, Sex	Reported patients (n)	Anatomy Involved	Duration
Dijk, 1973 [10]	61, female	1	Right ring finger	3 days
Deliss, 1982 [11]	55, female	6	Not reported	1-3 days
Eikenboom, 1991 [12]	61, female	2	Index and middle fingers	7 days
Layton, 1993 [13]	50, female	1	Middle finger	2 days
Parslew, 1995 [14]	44, female	2	Distal right palm	7 days
Kahira, 2001 [15]	49, female and male	11	Multiple digits of right and left hands	2-7 days
Robertson, 2002 [16]	36, female	2	Index and ring finger	2-3 months
Kampfen, 2005 [7]	60, female	1	Distal phalanx of the thumb	14 days
Huikeshoven, 2009 [17]	54, female	1	Right wrist	4 days
IchiToru, 2010 [18]	46, female	1	Left index and middle finger	Not reported
Mehmetia, 2010 [19]	57, female	1	Left middle finger	Not reported
Kluger, 2011 [20]	72, female	1	Right middle and ring finger	1 month
Weinberg, 2012 [21]	46, female	1	Right thumb and ring finger	Not reported
Thies, 2012 [22]	52, female	7	Multiple digits of right and left hands	Not reported
Harper, 2013 [23]	22, female	1	Left index finger	3 days
Echeverri, 2014 [24]	76, female	1	Thumb	3 days
Frerix, 2015 [25]	49, female	1	Middle finger	3 days
Füeßl, 2015 [26]	50, female	1	Left middle finger	2-3 days
Wollina, 2016 [27]	42, female	1	Right middle finger	Hours
Takeuchi, 2016 [5]	20, female	1	Left thumb and index finger	Not reported
Sigha, 2016 [28]	52, female	2	Right index finger	1-2 weeks
Watchorn, 2017 [29]	51, female	1	Palms of both hands	Several days
Jiménez, 2017 [1]	58, male	1	Right middle finger	Not reported
Yamamoto, 2017 [30]	69, female	1	Left ring finger	14 days
Ahmed, 2018 [31]	55, female	2	Left middle finger/Right index finger	7-10 days
Yamada, 2018 [32]	66, female	1	Right middle finger	7 days
Cohen, 2018 [33]	Not reported, male	1	Left thumb	5-15 minutes
Suzuki, 2019 [34]	30, female	1	Left ring finger	3 days
Notomi, 2019 [35]	40, male	1	Right middle finger	7 days
Godoy, 2019 [36]	81, female	1	Right middle finger	3 days
Yie, 2019 [9]	56, female	1	Right middle finger	7 days
Duvall, 2019 [37]	37, female	1	Right ring finger	Not reported
Pavlovic, 2019 [38]	51, female	1	Second toe	Not reported
Ada, 2019 [8]	47, female and male	24	Various digits of both hands	3-14 days
Ashrafzadeh, 2020 [39]	65, female	1	Left thumb and index finger	3-7 days
Kano, 2020 [40]	60, male	1	Left little finger	6 days
Castillo, 2020 [41]	48, female	1	Right middle finger	7 days
Helm, 2021 [42]	63, female	3	Right index, middle and ring fingers	1-3 days
Mizuno, 2021 [43]	78, male	1	Left index finger	7 days

## Conclusions

Achenbach’s syndrome is a benign skin manifestation with an unknown etiology. No specific treatment is required for this condition, and the skin lesions oftentimes recover spontaneously. Patients need to be reassured about the benign nature of the disease to avoid unnecessary investigations and prevent anxiety.
